# Evaluation of Severity of Illness Scores in the Pediatric ECMO Population

**DOI:** 10.3389/fped.2021.698120

**Published:** 2021-09-28

**Authors:** Venessa L. Pinto, Danielle Guffey, Laura Loftis, Melania M. Bembea, Philip C. Spinella, Sheila J. Hanson

**Affiliations:** ^1^Department of Pediatrics, Baylor College of Medicine, Houston, TX, United States; ^2^Institute for Clinical and Translational Research, Baylor College of Medicine, Houston, TX, United States; ^3^Department of Anesthesiology and Critical Care Medicine, Johns Hopkins University School of Medicine, Baltimore, MD, United States; ^4^Department of Pediatrics, Washington University School of Medicine, St. Louis, MO, United States; ^5^Department of Pediatrics, Medical College of Wisconsin, Milwaukee, WI, United States

**Keywords:** PIM2, PRISM III, PELOD, extracorporeal membrane oxygenation, severity of illness, pediatric

## Abstract

Though commonly used for adjustment of risk, severity of illness and mortality risk prediction scores, based on the first 24 h of intensive care unit (ICU) admission, have not been validated in the pediatric extracorporeal membrane oxygenation (ECMO) population. We aimed to determine the association of Pediatric Index of Mortality 2 (PIM2), Pediatric Risk of Mortality Score III (PRISM III) and Pediatric Logistic Organ Dysfunction (PELOD) scores with mortality in pediatric patients on ECMO. This was a retrospective cohort study of children ≤18 years of age included in the Pediatric ECMO Outcomes Registry (PEDECOR) from 2014 to 2018. Logistic regression and Receiver Operating Characteristics (ROC) curves were used to calculate the area under the curve (AUC) to evaluate association of mortality with the scores. Of the 655 cases, 289 (44.1%) did not survive until hospital discharge. AUCs for PIM2, PRISM III, and PELOD predicting mortality were 0.52, 0.52, and 0.51 respectively. PIM2, PRISM III, and PELOD scores are not associated with odds of mortality for pediatric patients receiving ECMO. These scores for a general pediatric ICU population should not be used for prognostication or risk stratification of a select population such as ECMO patients.

## Introduction

Tools to gauge severity of illness (SOI) and predict outcomes for children treated with extracorporeal membrane oxygenation (ECMO) can facilitate medical decision making, counseling of patients' families, and prognostication. ECMO is a cardio-pulmonary support modality for patients in severe cardiac and/or respiratory failure. It can be a life-saving therapy for patients who might otherwise not survive but is associated with significant morbidity and mortality. Patients requiring ECMO have increased severity of illness than the average pediatric intensive care unit (PICU) patient and have higher mortality (1).

Various scores are commonly used in PICUs worldwide for standardization of risk of mortality for both clinical benchmarking and for stratification of patient groups for research purposes. It is not uncommon to include markers of SOI or predictors of mortality in multivariable analysis to adjust for illness severity in comparing patients. These include Pediatric Index of Mortality 2 (PIM2), Pediatric Risk of Mortality Score III (PRISM III), and Pediatric Logistic Organ Dysfunction (PELOD) (2–4). While the validation set for these scores had an area under the curve (AUC) of >0.9 for outcome prediction, (2–4) they were calibrated for the general PICU population. They are calculated using data in the first 1 h (PIM2) or 24 h of PICU admission (PRISM III, PELOD) and significant variation in a patient's clinical course may exist from the time of score calculation on admission to when patients are cannulated onto ECMO. Illness severity scores validated for general PICU populations are sometimes used to adjust risk in specific populations, such as patients with hemophagocytic lymphohistiocytosis, those receiving renal replacement therapy, therapeutic plasma exchange, etc. (5–7). While these scores are also used in studies for pediatric patients on ECMO, there is however limited data on the ability of these scores to predict mortality in this select cohort. Knowing the validity of these illness severity scores in children receiving ECMO is needed to determine the appropriate of their use for clinical and research purposes in this cohort.

We sought to determine the association of PIM2, PRISM III, and PELOD scores calculated on admission to the PICU with mortality in pediatric patients treated with venovenous (VV) or venoarterial (VA) ECMO. We hypothesized that these scores would not be associated with outcomes for children receiving ECMO.

## Methods

We conducted a retrospective cohort study of patients in the Pediatric ECMO Outcomes Registry (PEDECOR) cannulated on ECMO from 2014 to 2018. PEDECOR is a web-based data platform, housed at Baylor College of Medicine's Dan L. Duncan Institute for Clinical and Translational Research. While initially built to support anticoagulation in ECMO research for BloodNet, a subgroup of PALISI (Pediatric Acute Lung Injury and Sepsis Investigators), it has expanded to include a robust clinical, laboratory and medication dataset for patients on ECMO. There were 10 U.S. children's hospitals participating in the registry. All participating institutions were trained on data entry; a data dictionary was provided, and two-person verification was undertaken at each site for interrater validation.

Baylor College of Medicine's Institutional Review Board approved the study protocol and waived the need for informed consent. We included all patients ≤ 18 years of age at the time of PICU admission. Children cannulated at an outside hospital and subsequently transferred to a PEDECOR site were excluded. We excluded patients in the registry prior to 2014 and those without mortality information. Patients that were missing all three scores were excluded from the study; we included patients with at least one of the three scores. For patients with multiple ECMO runs, only the first ECMO run was included in the analysis.

PIM2, PRISM III, and PELOD scores were directly added to the case report form if available to a study site, otherwise the individual variables comprising the score were directly entered into the online form from which the PIM2, PRISM III, and PELOD scores were then calculated. Other variables collected included demographic information such as sex and race, and clinical variables such as time from PICU admission to cannulation, weight, mode of ECMO cannulation, history of in-hospital cardiopulmonary arrest prior to ECMO, and ECPR which was defined as actively receiving CPR at the time of cannulation. The following variables were collected as markers of illness severity at the time of ECMO cannulation: urine output 8, 16, and 24 h prior to ECMO, highest lactate, total bilirubin, International Normalized Ratio (INR), platelet count, C-reactive protein (CRP), and creatinine in the 24 h prior to ECMO. OI was calculated within 6 h prior to cannulation, and VIS determined immediately prior to cannulation (8, 9).

### Statistical Analysis

Patient and clinical characteristics were summarized using mean with standard deviation, median with 25 and 75th percentiles, and frequency with percentage. The summary statistics were stratified by mortality and compared using two sample *t*-test, Wilcoxon rank sum test, Fisher's exact test, or Chi-square test, as appropriate. Logistic regression and Receiver Operating Characteristics (ROC) curves were used to calculate the area under the curve (AUC) for predicting mortality with PIM2, PRISM III, and PELOD to assess discrimination. The ROC curves were compared to each other. Calibration of the model based on the deciles of risk by the relevant score was performed using the Hosmer-Lemeshow test. Subgroup analyses were decided on a priori and included the VV, VA and ECPR cohorts, as well as early cannulation, defined as patients cannulated within the first 2 days of PICU admission. Statistical analyses were performed using Stata v 15.1 (Stata Corp, College Station, TX). For the calibration test a *p*-value >0.05 indicates a good model fit. For other analyses, a *p*-value of <0.05 was set as statistically significant *a priori*.

## Results

**Figure 1:**
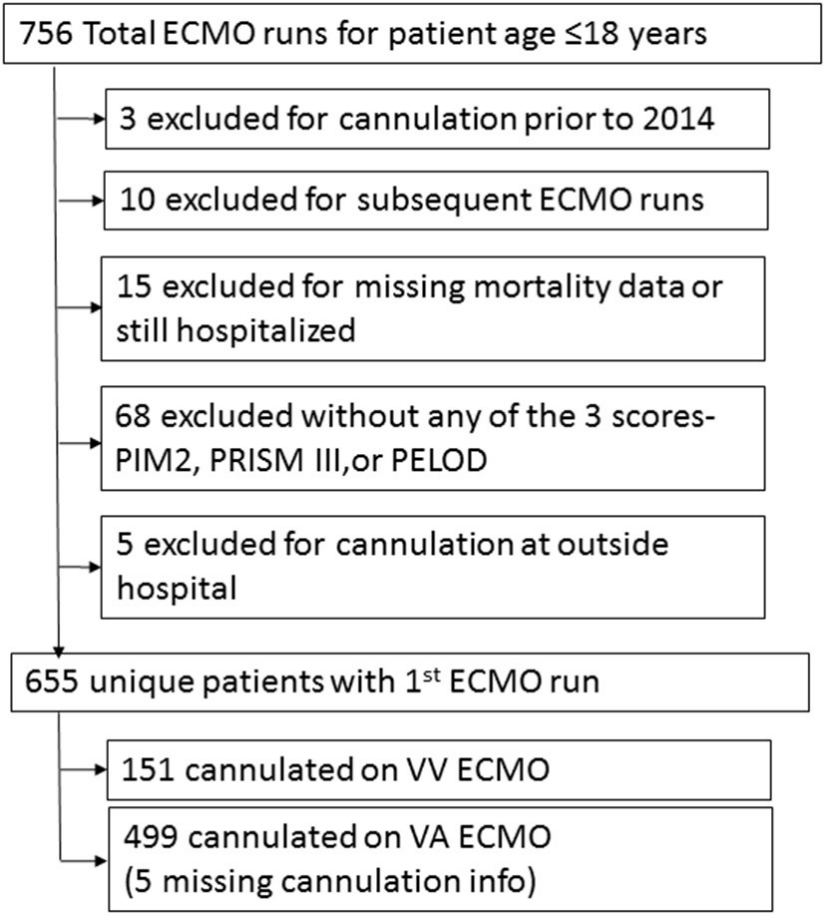
Study inclusion and exclusion flow diagram.

Seven hundred fifty six ECMO runs were evaluated in pediatric patients ≤18years. There were 655 patients in the registry that met inclusion and exclusion criteria (Figure 1). Prior to hospital discharge 289 (44.1%) patients died overall, with a mortality of 26.5% for VV cannulations and 49.1% for VA cannulations. Table 1 presents the summary statistics for patient and ECMO course characteristics by mortality. Table 2 presents the diagnostic indications for ECMO by survival and by cannulation type. Mortality was also higher for ECPR patients and those who received in-hospital CPR prior to ECMO.

**Table 1:** Summary statistics compared between survivors and non-survivors at time of discharge.

	**Alive (total** ***N*** **= 366)**			**Died (total** ***N*** **= 289)**			
**Variable**	***N***	***N*** **%, mean (SD) or**		***N***	***N*** **%, mean (SD) or**		***P*** **-value**
		**median (IQR)**			**median (IQR)**		
**Demographics**							
Age at ECMO (yr)^*^	366	0.3	(0.0,4.2)	289	0.3	(0.0,4.7)	0.374
Weight (kgs)^*^	318	5	(3.4,13.5)	251	4.6	(3.2,14.8)	0.401
Male gender	366	199	54.4%	289	155	53.6%	0.875
Race	360			278			0.33
American Indian or Alaska Native		1	0.3%		0	0%	
Asian		9	2.5%		12	4.3%	
Black or African American		99	27.5%		71	25.5%	
White		220	61.1%		162	58.3%	
Other		31	8.6%		33	11.9%	
**Cannulation type**	365			285			< 0.001
VV		111	30.4%		40	14%	
VA		254	69.6%		245	86%	
Time to cannulation (days)	366	1	(0.0,4.0)	289	2	(0.0,9.0)	0.013
**Pre-cannulation laboratory values**							
CRP (mg/dl)^*^	44	2.7	(0.5,9.4)	13	2.2	(0.8,6.5)	0.947
INR^*^	228	1.4	(1.2,1.9)	165	1.7	(1.3,2.2)	0.006
Highest arterial lactate (mmol/L)^*^							
Entire cohort	178	3	(1.4,8.1)	126	7.1	(2.3,16.1)	< 0.001
VA ECMO cohort	121	3.9	(2.1, 11.3)	107	9.5	(2.9, 17.5)	< 0.001
Highest creatinine (mg/dl)^*^	332	0.6	(0.4,0.8)	252	0.7	(0.5,1.0)	< 0.001
Platelets (k/mm3)^*^	276	167	(98.5,249.5)	208	127	(86.5,212.0)	0.003
Bilirubin (mg/dl)^*^	117	1.3	(0.4,3.4)	101	1.3	(0.5,3.3)	0.951
Hemoglobin (g/dl)^∧^	282	12.7	(2.7)	209	11.8	(3)	0.001
Urine output prior to ECMO (ml/kg/h)^*^							
8 h prior	292	0.4	(0.1,1.0)	228	0.3	(0.1,0.8)	0.012
16 h prior	285	1	(0.3,1.9)	227	0.8	(0.2,1.7)	0.071
24 h prior	280	1.4	(0.4,3.0)	230	1.2	(0.3,2.6)	0.203
**Severity of illness variables**							
Oxygenation index^*^							
Entire cohort	249	30	(6.0,48.0)	154	20.5	(5.0,45.0)	0.186
VV ECMO cohort	93	39	(23.0, 55.0)	37	43	(25.0, 62.0)	0.358
Vasoactive inotrope score^*^							
VA ECMO cohort	232	20.6	(8.5, 44.2)	226	25	(10.0, 75.0)	0.044
PIM2^∧^	363	−2.5	(1.9)	284	−2.2	(1.9)	0.088
PRISM III^∧^	352	14.7	(8.9)	278	15.9	(10.4)	0.117
PELOD^∧^	359	15.6	(10.8)	286	16.6	(13)	0.273
**CPR prior to ECMO**							
Entire cohort	365	72	19.7%	287	97	33.8%	< 0.001
VA ECMO cohort	253	65	25.7%	244	93	38.1%	0.004
*ECPR*							
Entire cohort	365	56	15.3%	286	102	35.7%	< 0.001
VA ECMO cohort	253	53	20.9%	243	100	41.2%	< 00.001

* *Median (25th, 75th percentiles)*.

∧ *Mean (SD)*.

*ECMO, extracorporeal membrane oxygenation; VV, venovenous; VA, venoarterial; CRP, C reactive protein; INR, international normalized ratio; PIM2 pediatric index of mortality 2; PRISM III, pediatric risk of mortality score III; PELOD, pediatric logistic organ dysfunction; CPR, cardiopulmonary resuscitation; ECPR, extracorporeal cardiopulmonary resuscitation*.

**Table 2:** Diagnostic indications for ECMO.

**Reason for ECMO**	**All patients**	**Alive**	**Dead**	**VV**	**VA**
ARDS due to trauma	3 (0.5%)	1 (0.3%)	2 (1%)	1 (1%)	2 (0.4%)
ARDS, other	121 (19%)	78 (22%)	43 (15%)	60 (41%)	61 (12%)
Arrhythmia	74 (11%)	28 (8%)	46 (16%)	2 (1%)	72 (15%)
Aspiration	25 (4%)	16 (5%)	9 (3%)	9 (6%)	16 (3%)
Cardiomyopathy-hypertrophic	5 (1%)	2 (1%)	3 (1%)	0	5 (1%)
Cardiomyopathy-restrictive	3 (0.5%)	0	3 (1%)	0	3 (1%)
Cardiomyopathy-dilated	33 (5%)	20 (6%)	13 (5%)	1 (1%)	32 (6%)
Congenital diaphragmatic hernia	68 (11%)	41 (11%)	27 (9%)	17 (12%)	50 (10%)
Congenital heart disease	186 (29%)	85 (24%)	101 (35%)	2 (1%)	181 (37%)
Coronary artery anomaly	10 (2%)	5 (1%)	5 (2%)	0	10 (2%)
Cystic fibrosis	1 (0.2%)	1 (0.3%)	0	1 (0.7%)	0
Drug intoxication	3 (0.5%)	2 (0.6%)	1 (0.4%)	0	3 (0.6%)
Hemophagocytic lymphohistiocytosis	1 (0.2%)	0	1 (0.4%)	0	1 (0.2%)
Hyperosmolar non-ketotic hyperglycemia	1 (0.2%)	1 (0.3%)	0	0	1 (0.2%)
Inborn error of metabolism	1 (0.2%)	1 (0.3%)	0	0	1 (0.2%)
Interstitial lung disease	6 (1%)	2 (1%)	4 (1%)	3 (2%)	3 (1%)
meconium aspiration	9 (1%)	9 (3%)	0	8 (5%)	1 (0.2%)
Myocarditis	26 (4%)	18 (5%)	8 (3%)	0	26 (5%)
Pneumonia	70 (11%)	48 (13%)	22 (8%)	51 (35%)	19 (4%)
Post-transplant graft failure—heart	18 (3%)	13 (4%)	5 (2%)	0	18 (4%)
Primary pulmonary hypertension	60 (9%)	38 (11%)	22 (8%)	9 (6%)	51 (10%)
Pulmonary embolus	3 (0.5%)	2 (0.6%)	1 (0.4%)	0	3 (0.6%)
Pulmonary hypertension (non-PPHN)	27 (4%)	15 (4%)	12 (4%)	8 (5%)	18 (4%)
Sepsis	61 (9%)	21 (6%)	40 (14%)	8 (5%)	53 (11%)
Status asthmaticus	19 (3%)	17 (5%)	2 (1%)	16 (11%)	3 (1%)
Submersion injury	6 (1%)	2 (1%)	4 (1%)	2 (1%)	3 (1%)

*Six hundred forty six patients had a reason for ECMO provided. Nine patients with a missing indication included one dead, eight alive, four VV, five VA*.

*N for all reasons for ECMO exceeds total number of cohort patients as some patients had more than one reason for ECMO listed*.

**Figure 2:**
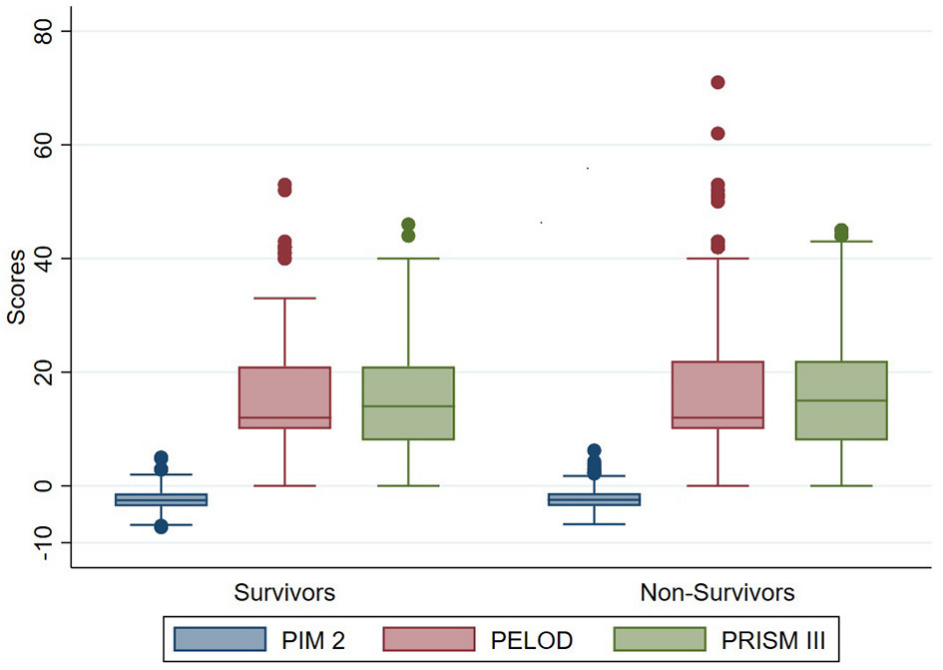
Score distributions for survivors and non-survivors.

**Figure 3:**
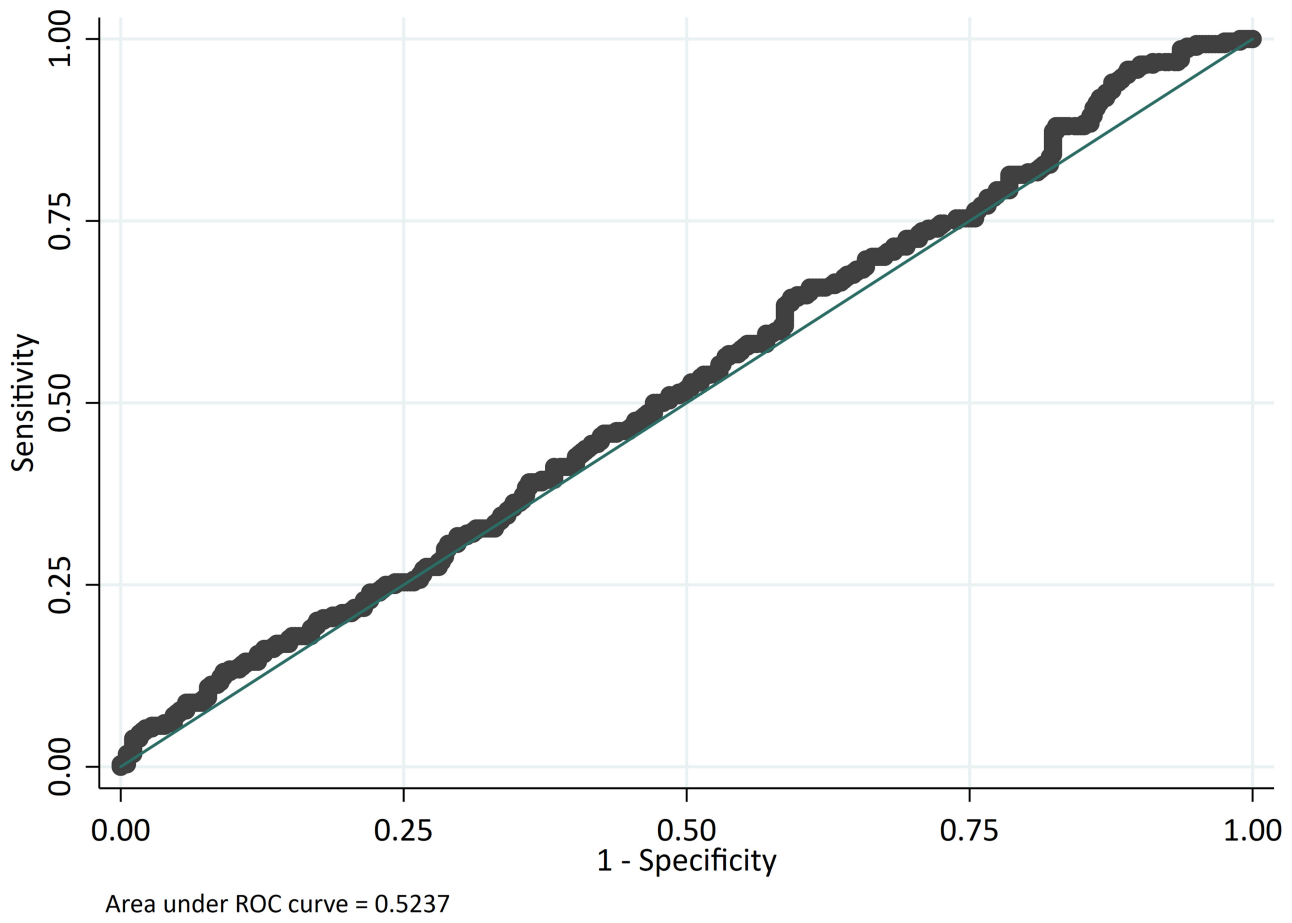
Receiver operating characteristic (ROC) curve and Logistic regression for Pediatric Index of Mortality 2 (PIM2) predicting mortality.

**Figure 4:**
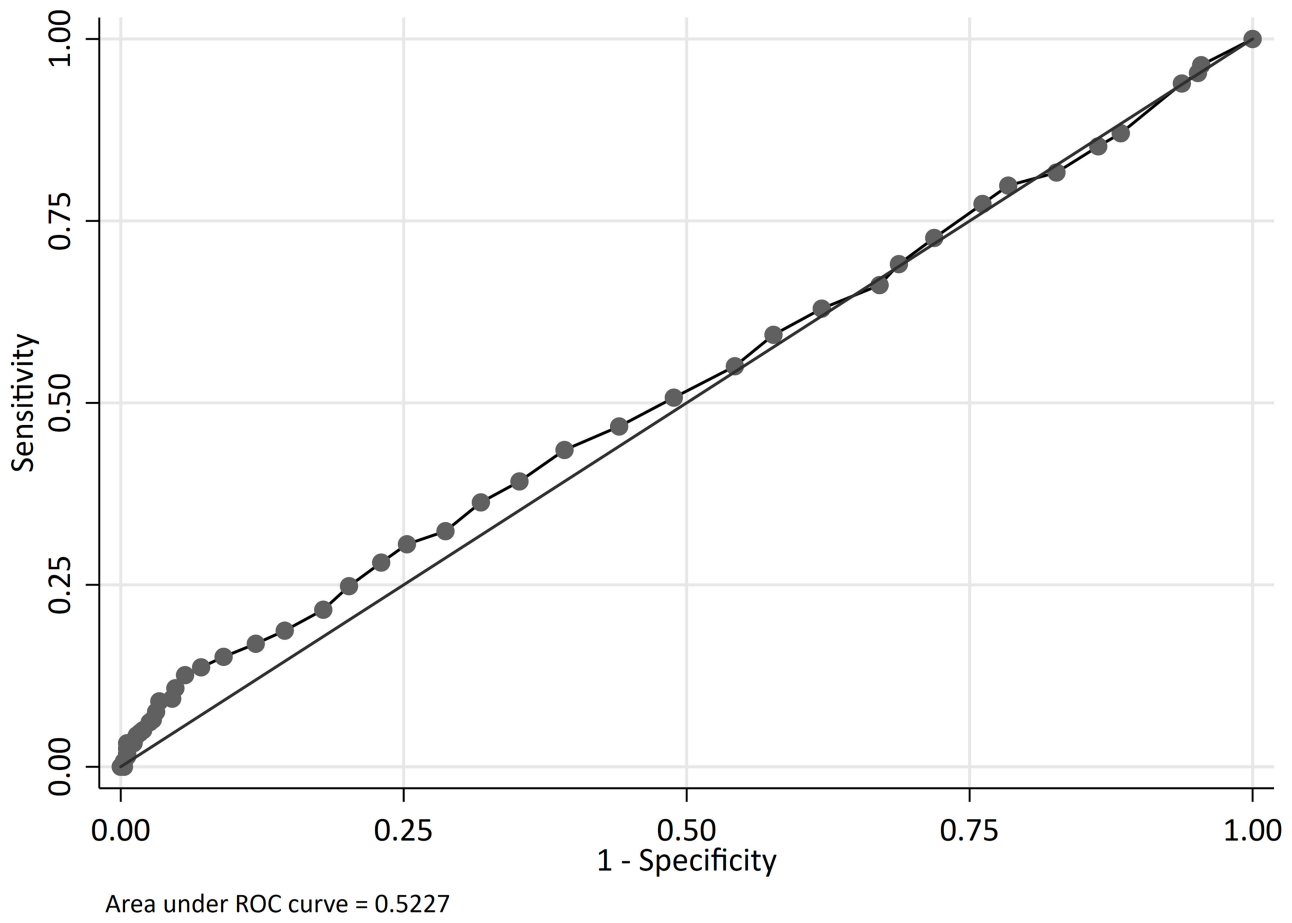
Receiver operating characteristic (ROC) curve and Logistic regression for Pediatric Risk of Mortality Score III (PRISM III) predicting mortality.

**Figure 5:**
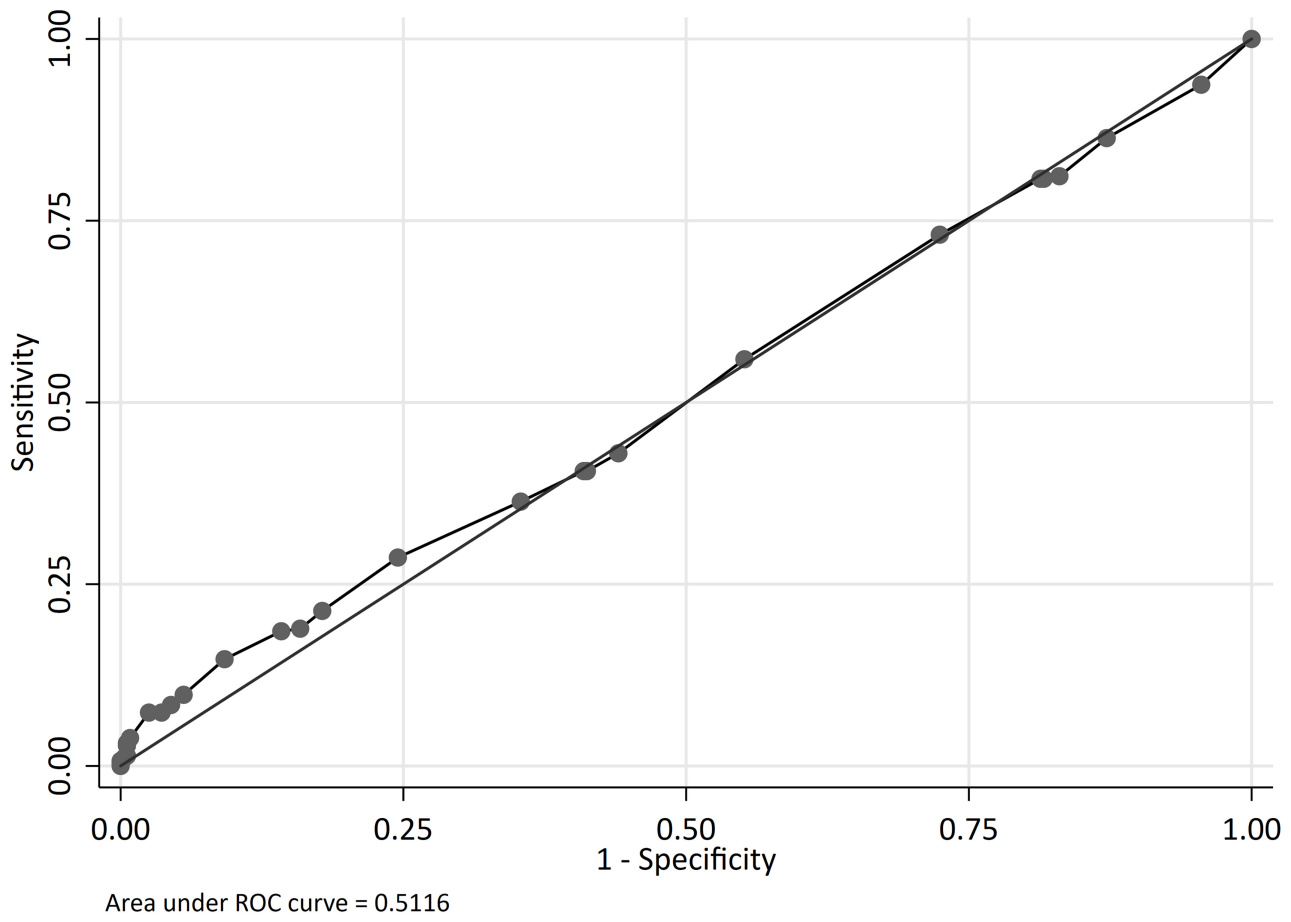
Receiver operating characteristic (ROC) curve and Logistic regression for Pediatric Logistic Organ Dysfunction (PELOD) predicting mortality.

The odds of mortality were not associated with PIM2, PRISM III, or PELOD scores (Table 3 and Figure 2). The AUC for PIM2 was 0.52 (Figure 3), for PRISM III was 0.52 (Figure 4) and for PELOD was 0.51 (Figure 5). There was no difference in the performance of these scores for patients on VV or VA ECMO (Supplementary Figures 1, 2). Subgroup analysis of early cannulation, showed that of the three scores, PRISM III performed slightly better at predicting mortality in this cohort with an AUC of 0.60 (Supplementary Figure 3). Table 4 presents the AUC and 95% confidence intervals for PIM2, PRISM III, and PELOD, with mortality for all patients, by cannulation type, and by timing of ECMO start. AUC's range from 0.47 up to 0.60 indicating poor discrimination. Hosmer-Lemeshow goodness of fit test indicates adequate calibration by score deciles for the entire cohort, the VV ECMO group, the VA ECMO group, and the group cannulated after 2 days. Calibration was poor among those cannulated earlier than 2 days, and for PELOD (*p* = 0.0195) but was adequate for PIM2 and PRISM III (Supplementary Table 1).

**Table 3:** Logistic regression and odds of mortality.

	**Odds ratio**	**95% Confidence interval**		***P*** **-value**
PIM2	1.07	0.99	1.17	0.088
PRISM III	1.01	1.00	1.03	0.118
PELOD	1.01	0.99	1.02	0.273

*PIM2, pediatric index of mortality 2; PRISM III, pediatric risk of mortality score III; PELOD, pediatric logistic organ dysfunction*.

**Table 4:** Area under the curve (AUC) for severity of illness scores predicting mortality.

**Variable**	**N**	**AUC**	**95% Confidence interval**	
**Entire cohort**				
PIM2	647	0.52	0.48	0.57
PRISM III	630	0.52	0.48	0.57
PELOD	645	0.51	0.47	0.56
**VV patients**				
PIM2	151	0.56	0.46	0.66
PRISM III	146	0.52	0.42	0.63
PELOD	147	0.52	0.41	0.62
**VA patients**				
PIM2	491	0.50	0.45	0.55
PRISM III	479	0.53	0.47	0.58
PELOD	493	0.52	0.46	0.57
**Early ECMO patients**				
PIM2	338	0.56	0.50	0.63
PRISM III	331	0.60	0.54	0.66
PELOD	335	0.56	0.50	0.62

*PIM2, pediatric index of mortality 2; PRISM III, pediatric risk of mortality score III; PELOD, pediatric logistic organ dysfunction; VV, venovenous; VA, venoarterial; ECMO, extracorporeal membrane oxygenation*.

*Early ECMO= Cannulation within 2 days of PICU admission*.

## Discussion

Multiple scores have been developed for the general PICU population for use in clinical bench marking and in research stratification by adjusting the expected risk of mortality for degree of illness. PIM2, a revised version of Pediatric Index of Mortality, is a risk of mortality score published in 2003 (3). It was developed using patient data gathered during the first hour of ICU admission from 12 units in Australia, New Zealand and the United Kingdom. PRISM III, a revision of the PRISM score, is a pediatric physiology-based score for mortality risk published in 1996. It was a U.S. study, validated using 17 clinical variables from the first 24 h of admission to the study site ICU (4). PELOD is a score for the assessment of multi-organ dysfunction, developed in 1999 and validated in 2003. It includes 10 variables corresponding to 5 organ dysfunctions with data collected from the first 24 h of admission to ICU (2). These scores have been validated in the general PICU population with AUC >0.9 for mortality outcome prediction. They have been used for mortality outcome prediction in specific groups of patients, such as those supported with ECMO, even though they were not originally created for this purpose.

Until recently, there were no data available on whether these three scores were appropriate for discriminating mortality risk in children treated with ECMO. Barbaro et al. showed that the PIM2, PRISM III, and PELOD scores had poor ability to discriminate mortality (10). The study was limited to 178 pediatric patients with respiratory failure on veno-venous ECMO. The validity of these scores in pediatric patients treated with veno-arterial ECMO for respiratory and cardiac failure was not studied. Our results, using a different data set, validate the previous finding that PIM2, PRISM III, and PELOD scores do not discriminate for death in pediatric patients receiving VV ECMO for respiratory failure, in a more diverse cohort across several institutions. Our study also shows that, in addition to VV patients, these scores have poor discrimination with mortality for patients receiving VA ECMO, as well as the overall larger cohort of 655 patients.

PIM2, PRISM III, and PELOD scores are based on parameters collected within the first few hours of admission to the PICU. PICU lengths of stay can range from days to even months, and a patient's clinical course can be highly variable influenced in part by the various therapies administered as well as individual patient responses to treatment. Cannulation for ECMO is often remote from admission, and scores calculated based on variables at the time of admission less accurately reflect the clinical state at the time of cannulation. We sought to determine if the scores performed better when applied to patients cannulated closer to the time the score was calculated. For the subgroup of patients cannulated within 2 days of PICU admission, 50% of study cohort, PRISM III had the highest AUC of 0.6 among the scores, which is far from ideal for a prognostic tool, and vastly inferior to the AUC of 0.9 for the overall PICU population. These SOI scores are not adequate for discrimination of mortality even when applied to patients who go on to receive ECMO cannulation closer to when the scores are calculated.

Severity of illness scores developed for the general PICU population are often used in research studies to compare patients based on illness severity. They have also been used to describe illness severity as well as risk of mortality in pediatric patients on ECMO. The PRISM score was used in the description of patients with meningococcal disease that were supported on ECMO (11) and in pediatric ECMO patients with acute respiratory failure (12). Even more recent studies such as a study on delirium in pediatric patients on VA ECMO in a cardiac ICU have used the PRISM III score to assess risk of mortality of patients in their cohort (13). PIM2, PELOD and PRISM scores were used in the baseline comparison of groups of patients who did not undergo tracheostomy vs. those that underwent an early or late tracheostomy after ECMO cannulation (14). Our study found that these scores cannot discriminate survivors from non-survivors after ECMO, hence they should not be used to compare baseline illness severity or to correlate outcomes. These general ICU scores used for risk stratification in focused populations are not valid for patients on ECMO.

## Limitations

Limitations of this study are variation of clinical practice among different centers, missing data, and limitation of analysis to the variables collected in the registry. The PIM2, PRISM III, and PELOD scores were contemporary at the start of the PEDECOR registry in 2013. While updated versions now exist for each of these scores, similar to the iterations of the scores used in this study, these recent iterations were again validated for a general pediatric ICU population, and again the score may be calculated remote to the cannulation date. This paper was limited to the performance of PIM2, PRISM III, and PELOD scores and did not evaluate all illness severity scores used in critically ill children. We did not seek to compare the performance of newer outcome prediction scores for children requiring ECMO for respiratory indications such as P-PREP (Pediatric Pulmonary Rescue With Extracorporeal Membrane Oxygenation Prediction Score) or PED-RESCUERS (Pediatric Risk Estimate Score for Children Using Extracorporeal Respiratory Support) (15, 16).

## Conclusion

Commonly used SOI scores- PIM2, PRISM III, and PELOD are not associated with mortality for pediatric patients receiving ECMO. This lack of discrimination persists for patients receiving VA, VV or those cannulated within 2 days of ICU admission. These scores should not be used by clinicians for medical decision making or prognostication for ECMO patients. They should not be used in research studies involving patients that receive ECMO to compare baseline illness severity between groups of patients.

## Data Availability Statement

The raw data supporting the conclusions of this article will be made available by the authors, without undue reservation.

## Ethics Statement

Baylor College of Medicine's Institutional Review Board reviewed and approved the studies involving human participants and waived the need for informed consent. Written informed consent from the participants' legal guardian/next of kin was not required to participate in this study in accordance with the national legislation and the institutional requirements.

## Author Contributions

VP, LL, and SH contributed to the conception and design of the study. DG performed the statistical analysis. VP wrote the first draft of the manuscript. LL, MB, and PS contributed to interpretation of the data and revising the manuscript. All authors contributed to manuscript revision, read, and approved the submitted version.

## Funding

MB's institution received funding from the National Institutes of Health (NIH)/National Institute of Neurological Disorders and Stroke and the Eunice Kennedy Shriver National Institute of Child Health and Human Development.

## Conflict of Interest

The authors declare that the research was conducted in the absence of any commercial or financial relationships that could be construed as a potential conflict of interest.

## Publisher's Note

All claims expressed in this article are solely those of the authors and do not necessarily represent those of their affiliated organizations, or those of the publisher, the editors and the reviewers. Any product that may be evaluated in this article, or claim that may be made by its manufacturer, is not guaranteed or endorsed by the publisher.

## Supplementary Material

Supplemental Figure 1A Receiver operating characteristic (ROC) curve and Logistic regression for PIM2 predicting mortality for Patients receiving Venovenous ECMO.

Supplemental Figure 1B Receiver operating characteristic (ROC) curve and Logistic regression for PRISM III predicting mortality for Patients receiving Venovenous ECMO.

Supplemental Figure 1C Receiver operating characteristic (ROC) curve and Logistic regression for PELOD predicting mortality for Patients receiving Venovenous ECMO.

Supplemental Figure 2A Receiver operating characteristic (ROC) curve and Logistic regression for PIM 2 predicting mortality for Patients receiving Venoarterial ECMO.

Supplemental Figure 2B Receiver operating characteristic (ROC) curve and Logistic regression for PRISM III predicting mortality for Patients receiving Venoarterial ECMO.

Supplemental Figure 2C Receiver operating characteristic (ROC) curve and Logistic regression for PELOD predicting mortality for Patients receiving Venoarterial ECMO.

Supplemental Figure 3A Receiver operating characteristic (ROC) curve and Logistic regression for PIM2 predicting mortality for Patients receiving early ECMO.

Supplemental Figure 3B Receiver operating characteristic (ROC) curve and Logistic regression for PRISM III predicting mortality for Patients receiving early ECMO.

Supplemental Figure 3C Receiver operating characteristic (ROC) curve and Logistic regression for PELOD predicting mortality for Patients receiving early ECMO.

## References

[1] Barbaro RP Paden ML Guner YS Raman L Ryerson LM Alexander P. Pediatric extracorporeal life support organization registry international report 2016. ASAIO J. (2017) 63:456–63. 10.1097/MAT.0000000000000603 28557863 PMC5626007

[2] Leteurtre S Martinot A Duhamel A Proulx F Grandbastien B Cotting J. Validation of the paediatric logistic organ dysfunction (PELOD) score: prospective, observational, multicentre study. Lancet. (2003) 362:192–7. 10.1016/S0140-6736(03)13908-6 12885479

[3] Slater A Shann F Pearson G. Paediatric index of mortality (PIM) study group. PIM2: a revised version of the paediatric index of mortality. Intensive Care Med. (2003) 29:278–85. 10.1007/s00134-002-1601-2 12541154

[4] Pollack MM Patel KM Ruttimann UE. PRISM III: an updated pediatric risk of mortality score. Crit Care Med. (1996) 24:743–52. 10.1097/00003246-199605000-00004 8706448

[5] Gregory J Greenberg J Basu S. Outcomes analysis of children diagnosed with hemophagocytic lymphohistiocytosis in the PICU. Pediatr Crit Care Med. (2019) 20:e185–90. 10.1097/PCC.0000000000001827 30520798

[6] Foland JA Fortenberry JD Warshaw BL Pettignano R Merritt RK Heard ML. Fluid overload before continuous hemofiltration and survival in critically ill children: a retrospective analysis. Crit Care Med. (2004) 32:1771–6. 10.1097/01.CCM.0000132897.52737.49 15286557

[7] Cortina G McRae R Chiletti R Butt W. Therapeutic plasma exchange in critically ill children requiring intensive care. Pediatr Crit Care Med. (2018) 19:e97–104. 10.1097/PCC.0000000000001400 29401139

[8] Trachsel D McCrindle BW Nakagawa S Bohn D. Oxygenation index predicts outcome in children with acute hypoxemic respiratory failure. Am J Respir Crit Care Med. (2005) 172:206–11. 10.1164/rccm.200405-625OC 15817802

[9] Gaies MG Gurney JG Yen AH Napoli ML Gajarski RJ Ohye RG. Vasoactive-inotropic score as a predictor of morbidity and mortality in infants after cardiopulmonary bypass. Pediatr Crit Care Med. (2010) 11:234–8. 10.1097/PCC.0b013e3181b806fc 19794327

[10] Barbaro RP Boonstra PS Kuo KW Selewski DT Bailly DK Stone CL. Evaluating mortality risk adjustment among children receiving extracorporeal support for respiratory failure. ASAIO J. (2019) 65:277–84. 10.1097/MAT.0000000000000813 29746311

[11] Goldman AP Kerr SJ Butt W Marsh MJ Murdoch IA Paul T. Extracorporeal support for intractable cardiorespiratory failure due to meningococcal disease. Lancet. (1997) 349:466–9. 10.1016/S0140-6736(96)12106-1 9040577

[12] Green TP Timmons OD Fackler JC Moler FW Thompson AE Sweeney MF. The impact of extracorporeal membrane oxygenation on survival in pediatric patients with acute respiratory failure. Pediatric Critical Care Study Group. Crit Care Med. (1996) 24:323–9. 10.1097/00003246-199602000-00023 8605808

[13] Patel AK Biagas KV Clark EC Traube C. Delirium in the pediatric cardiac extracorporeal membrane oxygenation patient population: a case series. Pediatr Crit Care Med. (2017) 18:e621–24. 10.1097/PCC.0000000000001364 29076929

[14] Tripathi S Swayampakula AK Deshpande GG Astle M Wang Y Welke KF. Illustration of the current practice and outcome comparison of early versus late tracheostomy after pediatric ECMO. Int J Artif Organs. (2020) 43:726–34. 10.1177/0391398820913571 32228203

[15] Bailly DK Reeder RW Zabrocki LA Hubbard AM Wilkes J Bratton SL. Development and validation of a score to predict mortality in children undergoing extracorporeal membrane oxygenation for respiratory failure: pediatric pulmonary rescue with extracorporeal membrane oxygenation prediction score. Crit Care Med. (2017) 45:e58–e66. 10.1097/CCM.0000000000002019 27548818 PMC5532876

[16] Barbaro RP Boonstra PS Paden ML Roberts LA Annich GM Bartlett RH. Development and validation of the pediatric risk estimate score for children using extracorporeal respiratory support (Ped-RESCUERS). Intensive Care Med. (2016) 42:879–88. 10.1007/s00134-016-4285-8 27007109 PMC6379065

