# Supplementary figures and images for: Evaluation of Severity of Illness Scores in the Pediatric ECMO Population
Source: Front Pediatr. 2021 Sep 28;9:698120. doi: 10.3389/fped.2021.698120 (PMC8506160; doi:10.3389/fped.2021.698120)

## Slide 1
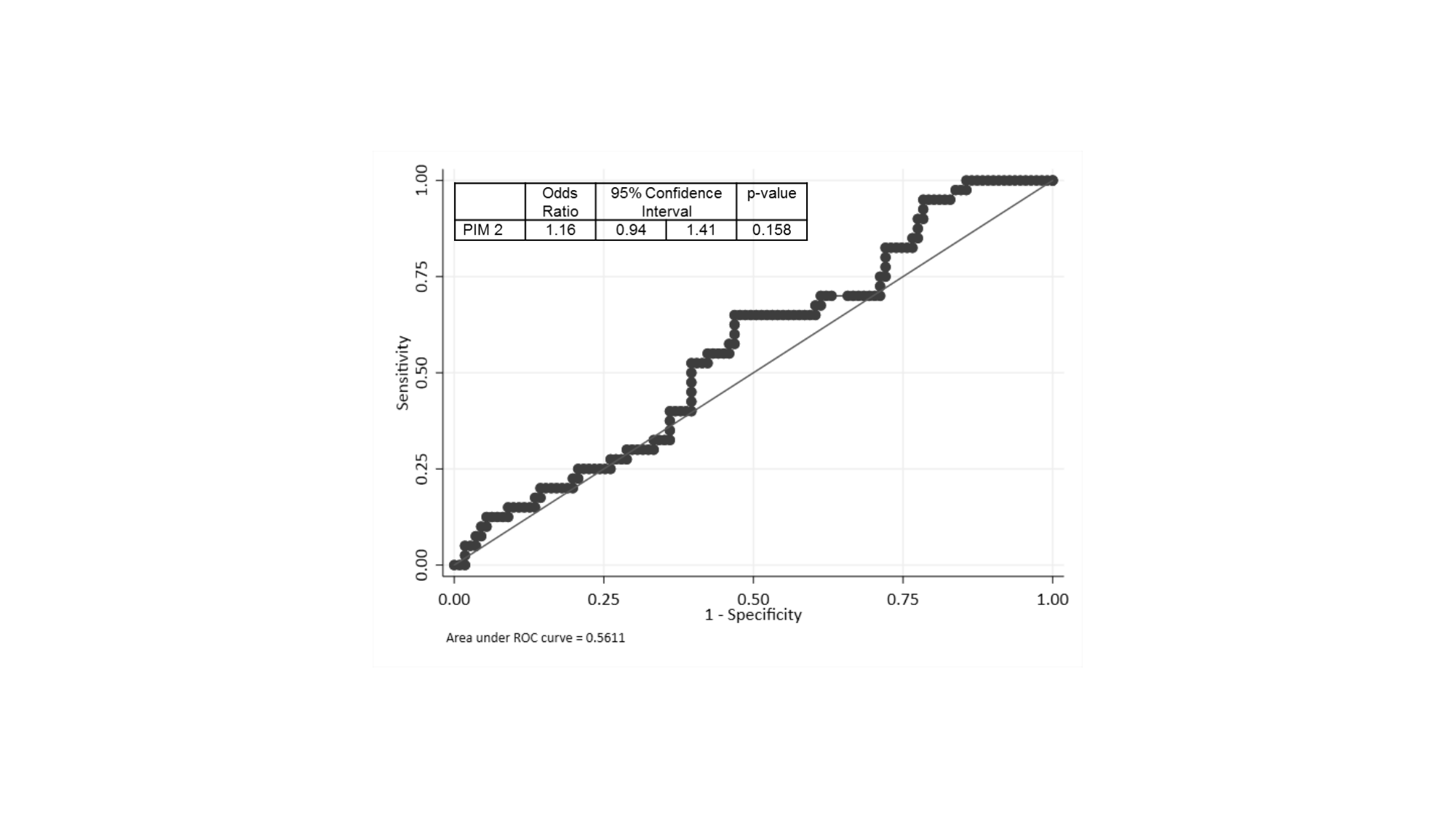

Supplement: Supplemental Figure 1A — Receiver operating characteristic (ROC) curve and Logistic regression for PIM2 predicting mortality for Patients receiving Venovenous ECMO. [file Data_Sheet_1.zip › Pinto_SupplementalFigure1a.pptx]

## Slide 1
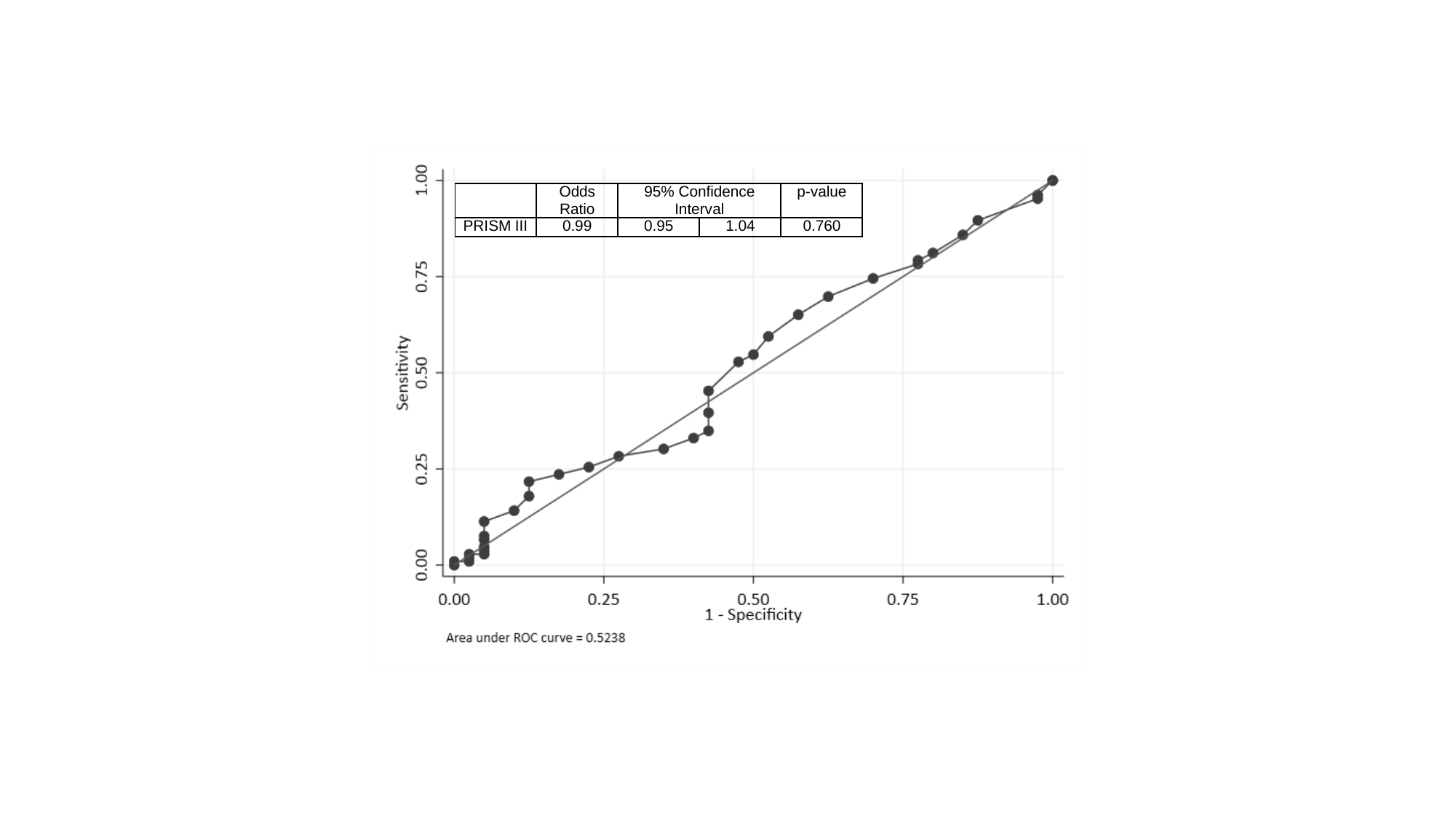

| | Odds Ratio | 95% Confidence Interval | | p-value |
| --- | --- | --- | --- | --- |
| PRISM III | 0.99 | 0.95 | 1.04 | 0.760 |

Supplement: Supplemental Figure 1A — Receiver operating characteristic (ROC) curve and Logistic regression for PIM2 predicting mortality for Patients receiving Venovenous ECMO. [file Data_Sheet_1.zip › Pinto_SupplementalFigure1b.pptx]

## Slide 1
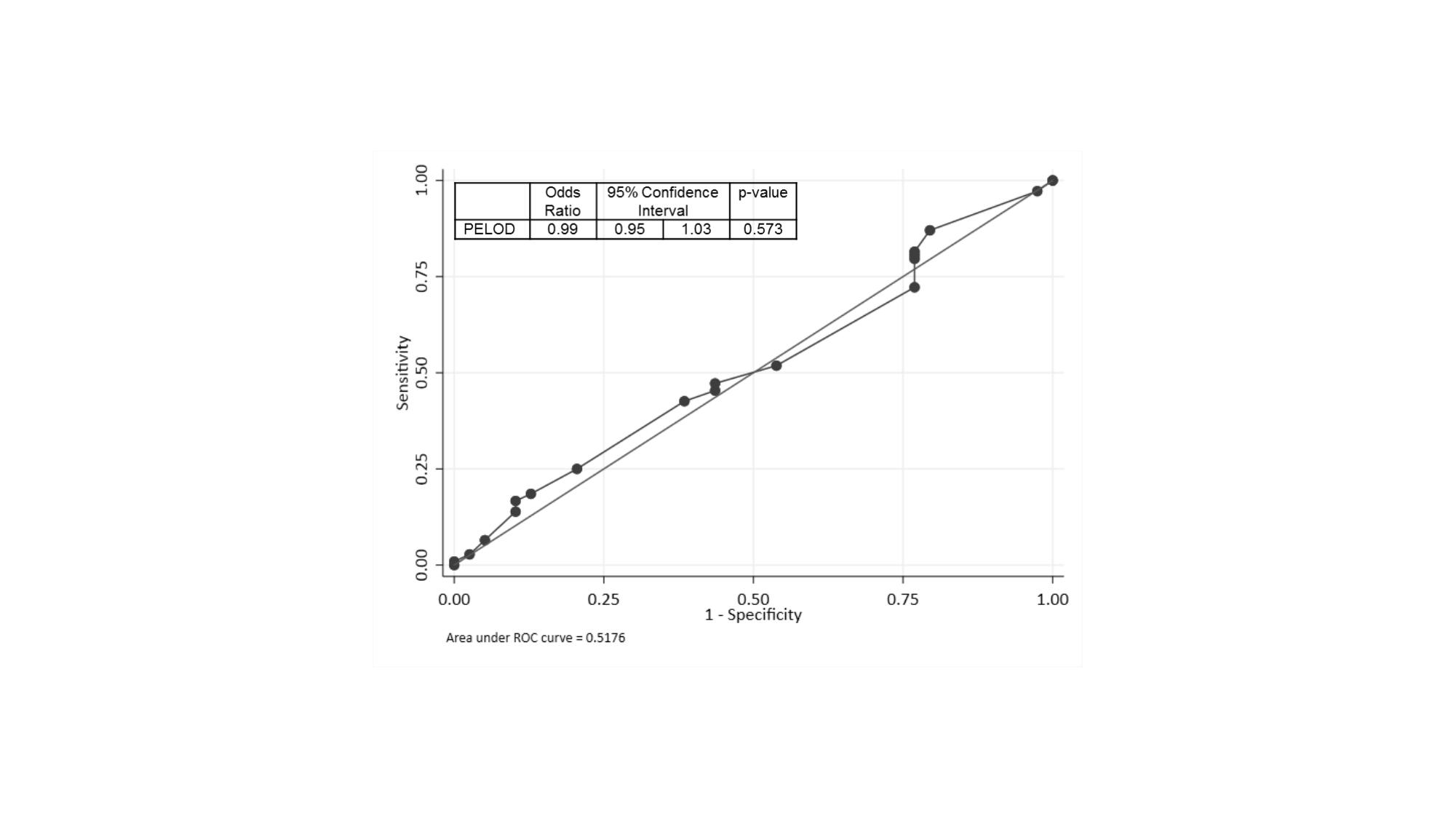

Supplement: Supplemental Figure 1A — Receiver operating characteristic (ROC) curve and Logistic regression for PIM2 predicting mortality for Patients receiving Venovenous ECMO. [file Data_Sheet_1.zip › Pinto_SupplementalFigure1c.pptx]

## Slide 1
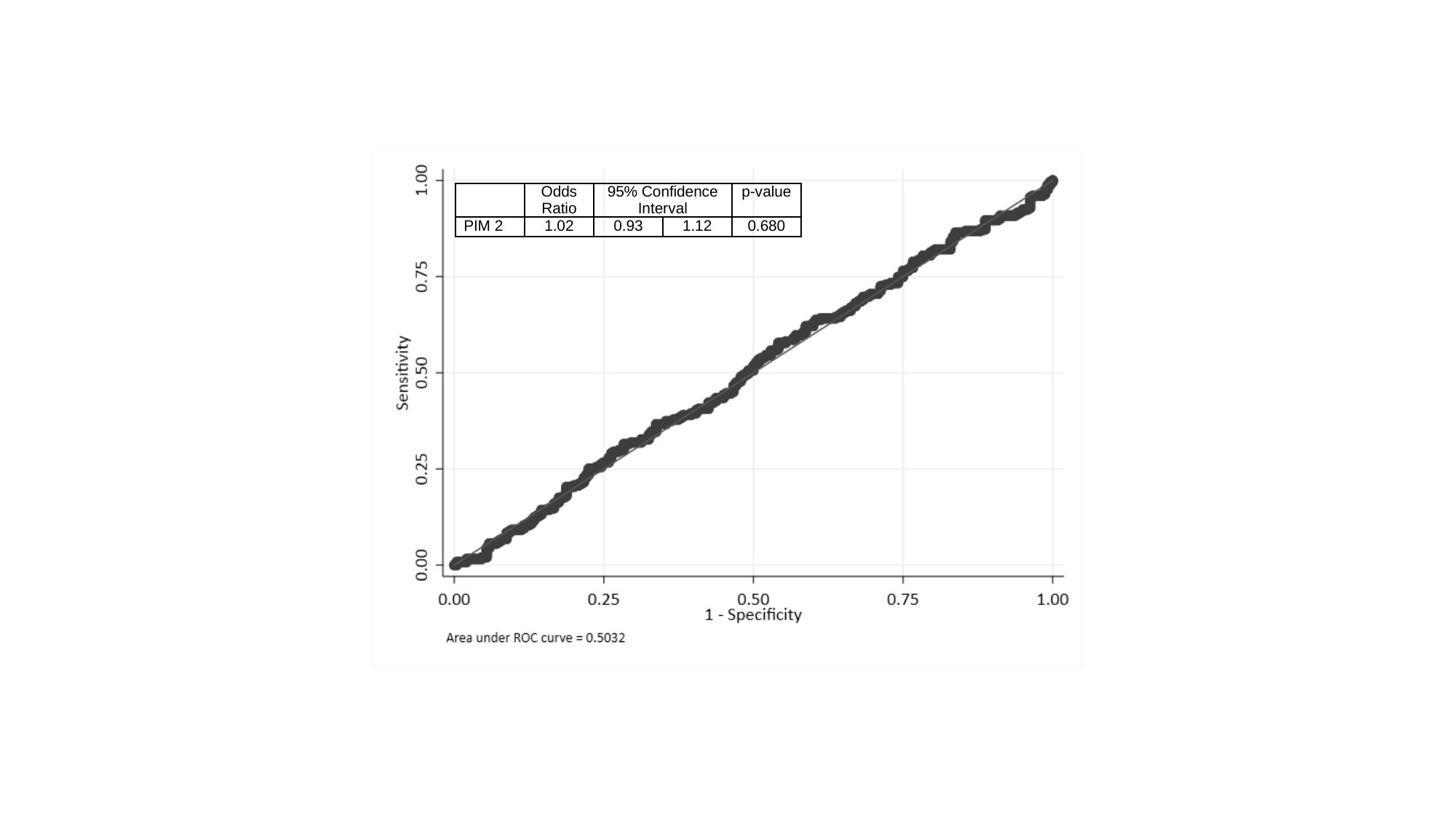

| | Odds Ratio | 95% Confidence Interval | | p-value |
| --- | --- | --- | --- | --- |
| PIM 2 | 1.02 | 0.93 | 1.12 | 0.680 |

Supplement: Supplemental Figure 1A — Receiver operating characteristic (ROC) curve and Logistic regression for PIM2 predicting mortality for Patients receiving Venovenous ECMO. [file Data_Sheet_1.zip › Pinto_SupplementalFigure2a.pptx]

## Slide 1
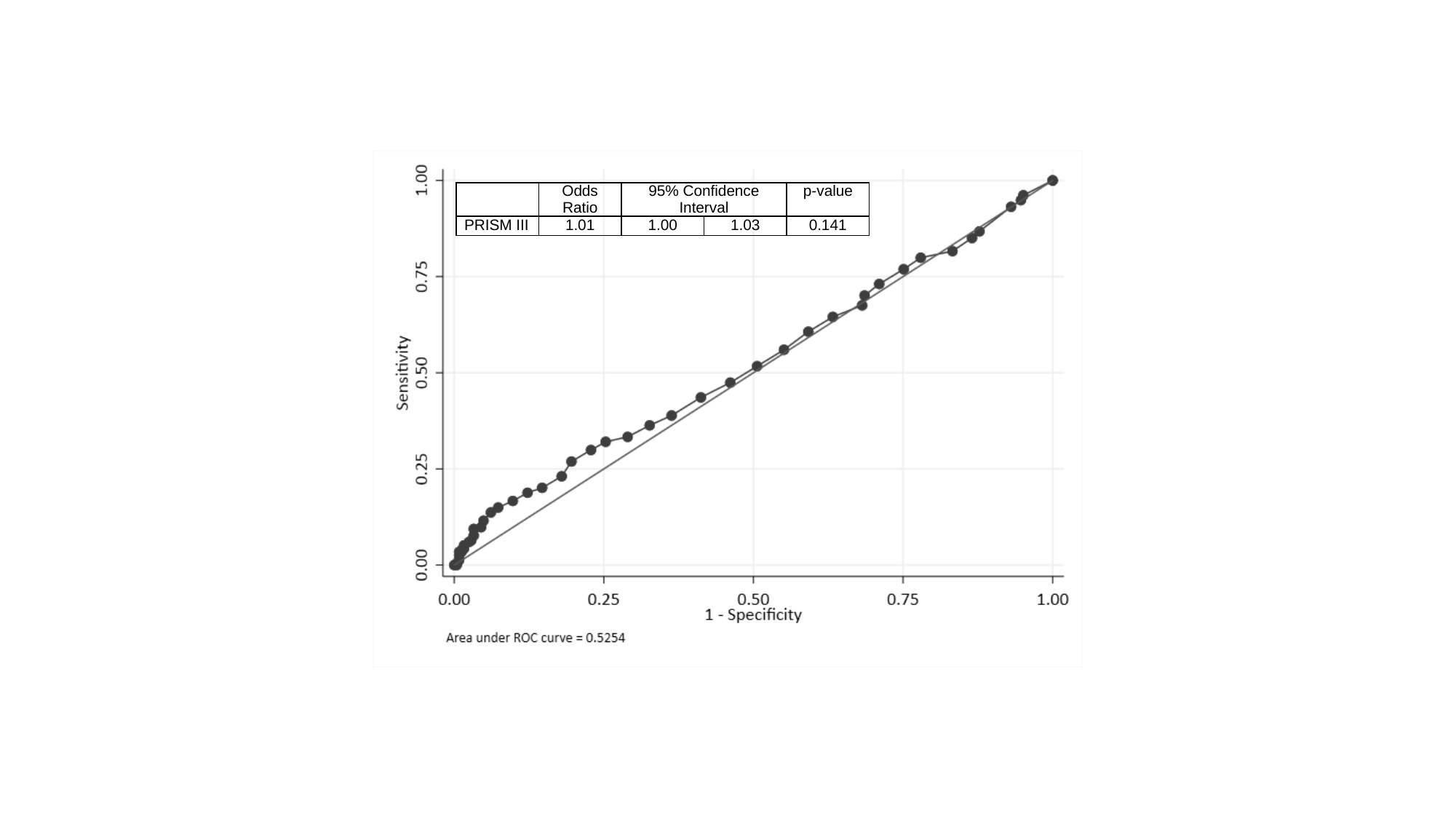

| | Odds Ratio | 95% Confidence Interval | | p-value |
| --- | --- | --- | --- | --- |
| PRISM III | 1.01 | 1.00 | 1.03 | 0.141 |

Supplement: Supplemental Figure 1A — Receiver operating characteristic (ROC) curve and Logistic regression for PIM2 predicting mortality for Patients receiving Venovenous ECMO. [file Data_Sheet_1.zip › Pinto_SupplementalFigure2b.pptx]

## Slide 1
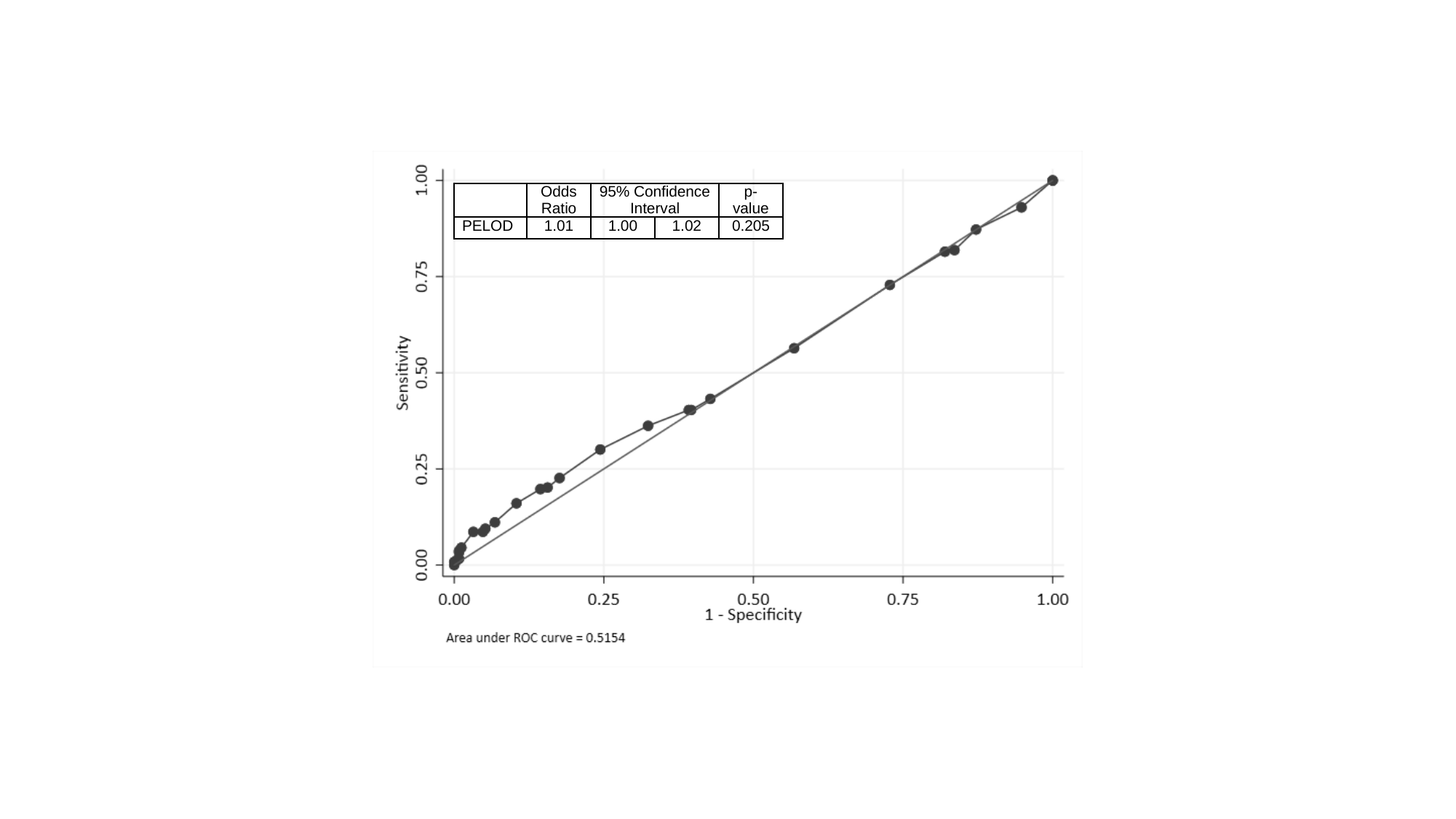

| | Odds Ratio | 95% Confidence Interval | | p-value |
| --- | --- | --- | --- | --- |
| PELOD | 1.01 | 1.00 | 1.02 | 0.205 |

Supplement: Supplemental Figure 1A — Receiver operating characteristic (ROC) curve and Logistic regression for PIM2 predicting mortality for Patients receiving Venovenous ECMO. [file Data_Sheet_1.zip › Pinto_SupplementalFigure2c.pptx]

## Slide 1
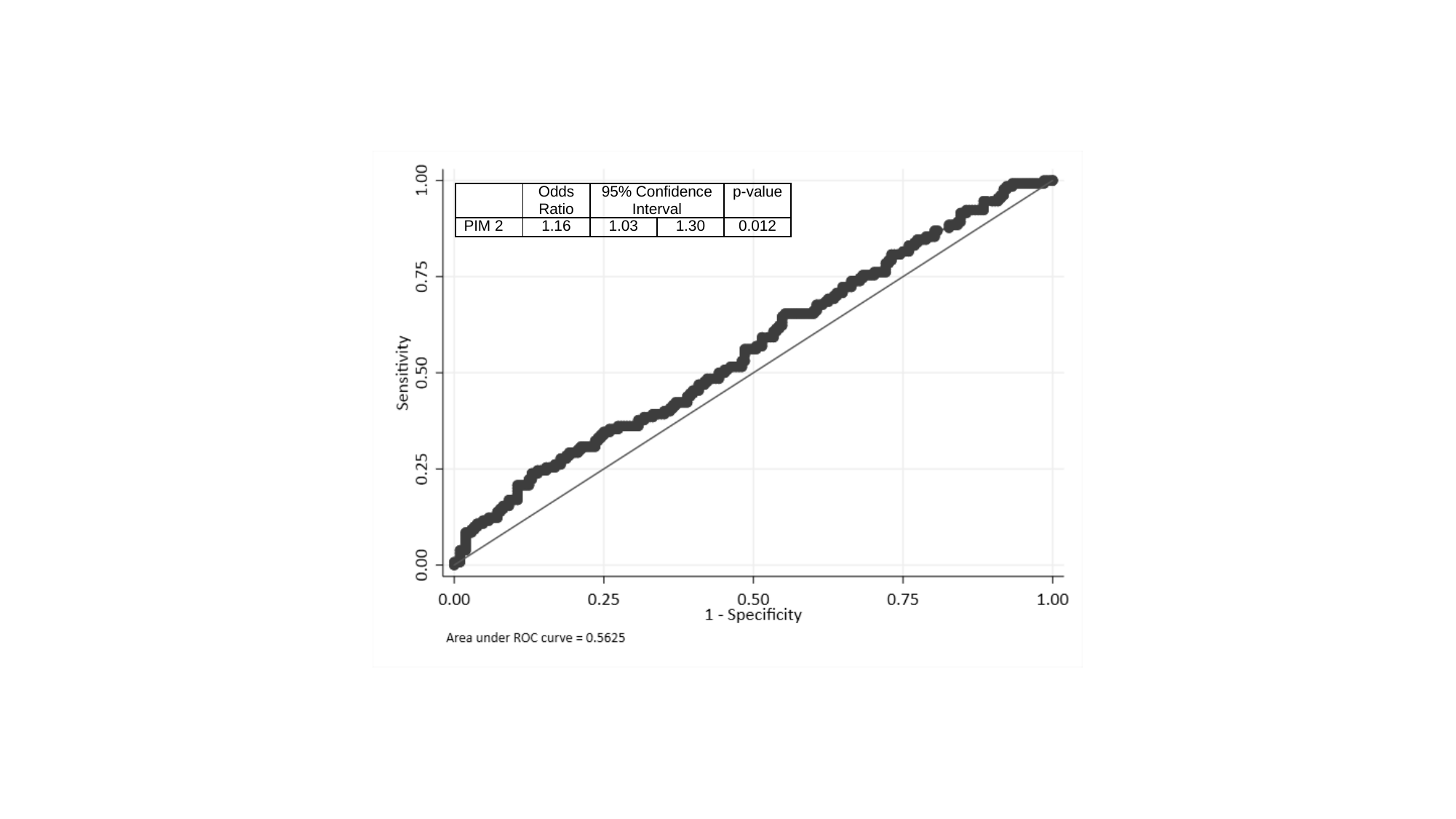

| | Odds Ratio | 95% Confidence Interval | | p-value |
| --- | --- | --- | --- | --- |
| PIM 2 | 1.16 | 1.03 | 1.30 | 0.012 |

Supplement: Supplemental Figure 1A — Receiver operating characteristic (ROC) curve and Logistic regression for PIM2 predicting mortality for Patients receiving Venovenous ECMO. [file Data_Sheet_1.zip › Pinto_SupplementalFigure3a.pptx]

## Slide 1
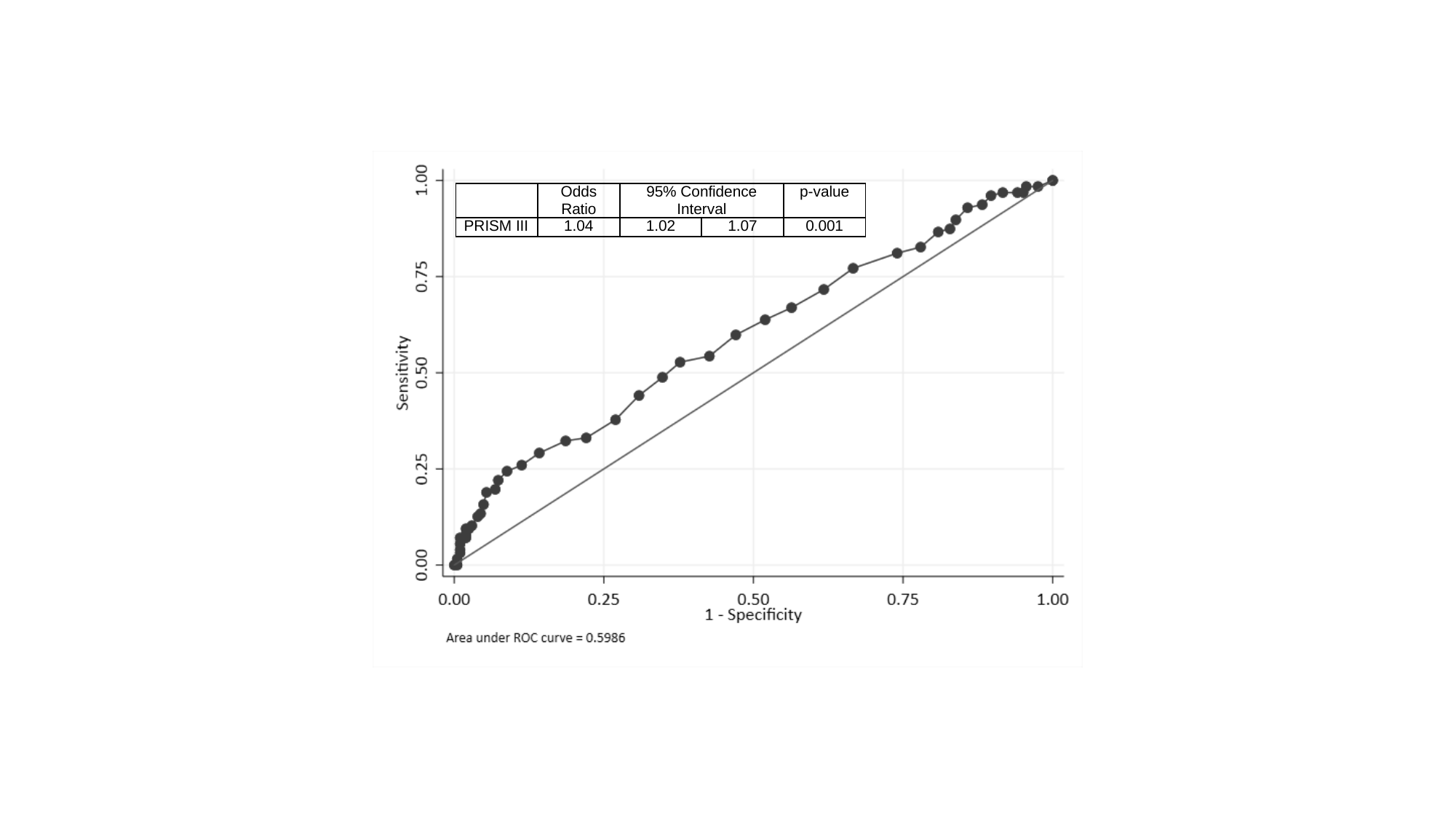

| | Odds Ratio | 95% Confidence Interval | | p-value |
| --- | --- | --- | --- | --- |
| PRISM III | 1.04 | 1.02 | 1.07 | 0.001 |

Supplement: Supplemental Figure 1A — Receiver operating characteristic (ROC) curve and Logistic regression for PIM2 predicting mortality for Patients receiving Venovenous ECMO. [file Data_Sheet_1.zip › Pinto_SupplementalFigure3b.pptx]

## Slide 1
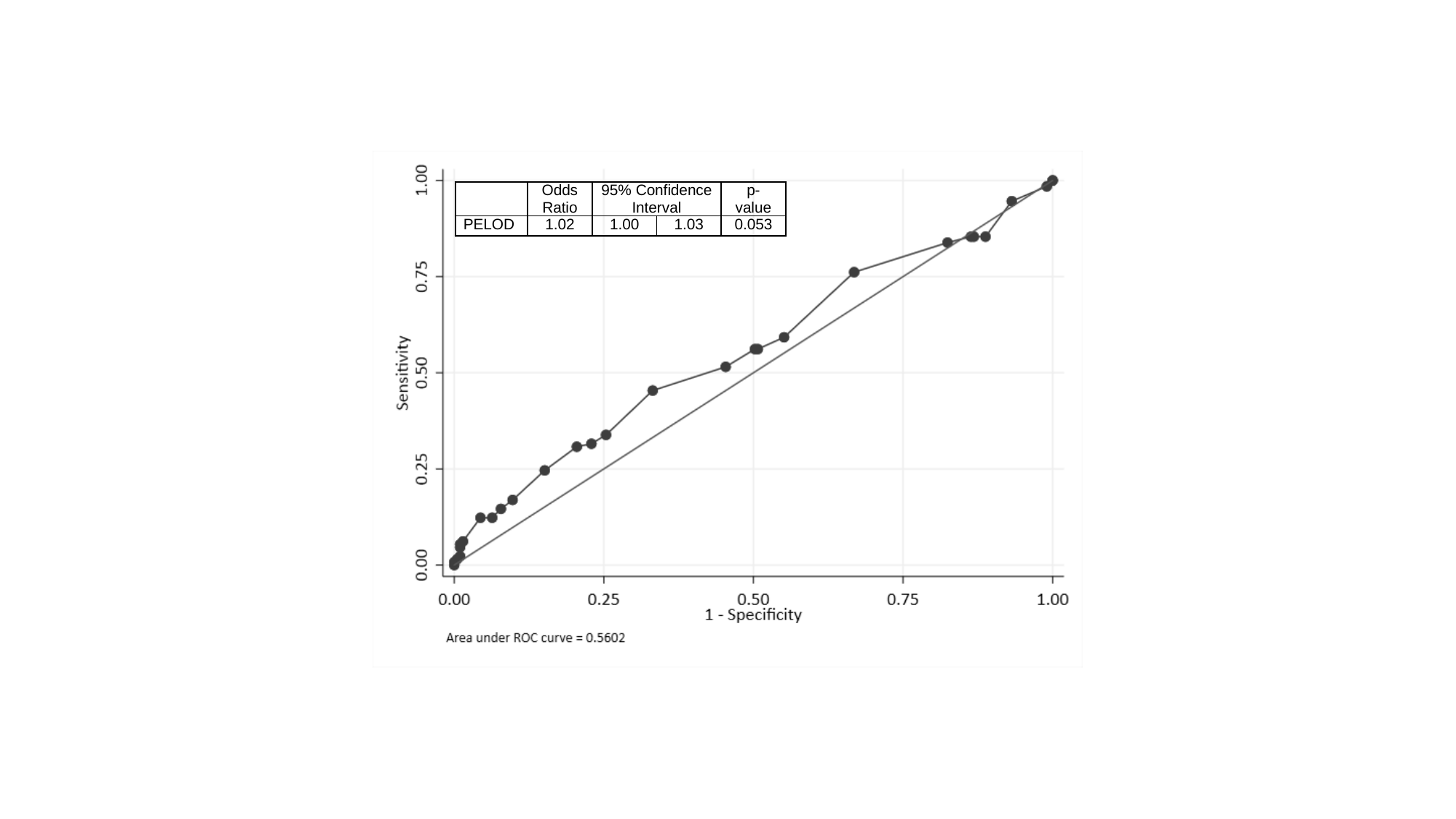

| | Odds Ratio | 95% Confidence Interval | | p-value |
| --- | --- | --- | --- | --- |
| PELOD | 1.02 | 1.00 | 1.03 | 0.053 |

Supplement: Supplemental Figure 1A — Receiver operating characteristic (ROC) curve and Logistic regression for PIM2 predicting mortality for Patients receiving Venovenous ECMO. [file Data_Sheet_1.zip › Pinto_SupplementalFigure3c.pptx]
