# Supplementary material for: Evaluation of Severity of Illness Scores in the Pediatric ECMO Population
Source: Front Pediatr. 2021 Sep 28;9:698120. doi: 10.3389/fped.2021.698120 (PMC8506160; doi:10.3389/fped.2021.698120)
Supplement: Supplementary file 2 [file Table_1.docx]

| Model | Prob of death | Death across deciles | | Hosmer-Lemeshow goodness of fit test | |
| --- | --- | --- | --- | --- | --- |
|  |  | Observed | Expected | Chi-square | P-value |
| PIM 2 | | | | 4.86 | 0.7721 |
| Group 1 | 0.4215 | 25 (33.8%) | 31.2 (42.1%) |  |  |
| Group 2 | 0.4225 | 29 (50.0%) | 24.5 (42.2%) |  |  |
| Group 3 | 0.4242 | 27 (42.9%) | 26.7 (42.3%) |  |  |
| Group 4 | 0.4262 | 29 (44.6%) | 27.6 (42.5%) |  |  |
| Group 5 | 0.4287 | 31 (44.9%) | 29.5 (42.7 %) |  |  |
| Group 6 | 0.4336 | 26 (43.3%) | 25.9 (43.1%) |  |  |
| Group 7 | 0.4391 | 30 (45.5%) | 28.8 (43.6%) |  |  |
| Group 8 | 0.4486 | 28 (44.4%) | 28.0 (44.4%) |  |  |
| Group 9 | 0.4719 | 26 (40.0%) | 29.8 (45.9%) |  |  |
| Group 10 | 0.5315 | 33 (51.6%) | 32.1 (50.1%) |  |  |
| PRISM 3 | | | | 9.35 | 0.3133 |
| Group 1 | 0.402 | 36 (46.8%) | 30.7 (39.8%) |  |  |
| Group 2 | 0.4115 | 20 (36.4%) | 22.5 (40.9%) |  |  |
| Group 3 | 0.421 | 30 (46.9%) | 26.8 (41.8%) |  |  |
| Group 4 | 0.4306 | 27 (40.9%) | 28.2 (42.8%) |  |  |
| Group 5 | 0.437 | 24 (43.6%) | 24.0 (43.6%) |  |  |
| Group 6 | 0.4467 | 32 (40.0%) | 35.5 (44.3%) |  |  |
| Group 7 | 0.4564 | 24 (40.7%) | 26.7 (45.3%) |  |  |
| Group 8 | 0.4662 | 25 (49.0%) | 23.6 (46.3%) |  |  |
| Group 9 | 0.4809 | 22 (36.7%) | 28.4 (47.4%) |  |  |
| Group 10 | 0.5413 | 38 (60.3%) | 31.7 (50.3%) |  |  |
| PELOD | | | | 6.38 | 0.4963 |
| Group 1 | 0.4163 | 39 (45.9%) | 35.3 (41.6%) |  |  |
| Group 2 | 0.4325 | 38 (41.8%) | 38.8 (42.7%) |  |  |
| Group 3 | 0.4343 | 49 (44.1%) | 48.2 (43.4%) |  |  |
| Group 4 | 0.4361 | 37 (48.1%) | 33.6 (43.6%) |  |  |
| Group 5 | 0.4506 | 19 (38.0%) | 22.3 (44.6%) |  |  |
| Group 6 | 0.4524 | 22 (36.1%) | 27.6 (45.2%) |  |  |
| Group 7 | 0.4542 | 21 (46.7%) | 20.4 (45.4%) |  |  |
| Group 8 | 0.4724 | 33 (42.9%) | 36.1 (46.8%) |  |  |
| Group 9 | 0.5438 | 28 (58.3%) | 23.6 (49.3%) |  |  |

Supplemental Table 1: Calibration assessment using Hosmer-Lemeshow test utilizing deciles of risk
